# A Giant Parathyroid Adenoma Presenting as Coughs and Dyspnea in a Young Woman: A Case Report

**DOI:** 10.1002/ccr3.71500

**Published:** 2025-12-29

**Authors:** Somayeh Chahkandi, Nasrin Amiri‐Dashatan, Mehdi Koushki, Mojgan Sanjari

**Affiliations:** ^1^ Zanjan Metabolic Diseases Research Center Zanjan University of Medical Sciences Zanjan Iran; ^2^ Cancer Gene Therapy Research Center Zanjan University of Medical Sciences Zanjan Iran; ^3^ Department of Clinical Biochemistry, School of Medicine Zanjan University of Medical Sciences Zanjan Iran; ^4^ Endocrinology and Metabolism Research Center Institute of Basic and Clinical Physiology Sciences, Kerman University of Medical Sciences Kerman Iran

**Keywords:** Giant parathyroid adenoma (GPA), hypercalcemia, parathyroid hormone (PTH), primary hyperparathyroidism (PHPT)

## Abstract

A parathyroid adenoma is defined as a benign tumor in the parathyroid glands. A type of parathyroid adenoma is giant parathyroid adenoma that weighs > 3.5 g and has a size of more than 2 cm. A 37‐year‐old woman presented with coughs and dyspnea without fever, hemoptysis, and weight loss. Examination of the patient revealed a diffusely enlarged thyroid gland and a positive Pemberton's sign. On the CT scan, the hypodense mass in the superior mediastinum was noted with tracheal compression. The resected mass weighed 15 g, and histopathology indicated a giant parathyroid adenoma (GPA). Giant parathyroid adenoma, as a rare cause of hyperparathyroidism, is diagnosed by imaging and laboratory methods. This case showed that the differential diagnosis of GPA is necessary and should be considered. In addition, the main method of treatment for this disease is surgery and removal of the parathyroid adenoma.

## Summary

- A clinician should be specially considered the symptoms such as coughs and dyspnea in giant parathyroid adenoma in comparison with parathyroid adenoma regarding to be larger of gland and more production of calcium.
- These symptoms may be different depended on to be male and female.

**Abbreviations**
FNA: Fine needle aspirate
GPA: Giant Parathyroid Adenoma
HPF: High‐Power Field
PC: Parathyroid Carcinoma
PHPT: Primary Hyperparathyroidism
PTH: Parathyroid Hormone
RBC: red blood cell

## 1 Introduction

The parathyroid glands are situated posterior to the thyroid gland, with each gland weighing approximately 60 mg. These glands secrete parathyroid hormone (PTH), which plays a crucial role in regulating calcium metabolism. Primary hyperparathyroidism (PHPT) is a common endocrine disorder characterized by a condition in which one or more parathyroid glands are overactive, leading to excessive release of parathyroid hormone (PTH) and hypercalcemia [1, 2]. It is the most common parathyroid disorder and also the third most common endocrine disorder. The prevalence of PHPT in the general population is 25 per 100,000 [3]. Causes of hyperparathyroidism include a solitary parathyroid adenoma in about 85% of patients, hyperplasia in 15%, multiple adenomas or hyperplasia of all four glands in 5%–10%, and parathyroid cancer in less than 1% of patients [4, 5, 6].

Primary hyperparathyroidism is sporadic in 90%–95% of cases and familial in 5%–10% of cases [7]. This disease is more common in postmenopausal women with a ratio of 3–4:1 in females to males [8]. The normal weight of the parathyroid gland is 60 mg, whereas in the condition of giant parathyroid adenoma (GPA), its weight reaches more than 3.5 g [9, 10].

New diagnostic methods, such as biochemical and imaging techniques, have made it possible to recognize parathyroid adenoma even in the asymptomatic stage. Hypercalcemia, hypophosphatemia, and elevated PTH are the characteristic features and pathophysiology of GPA. The elevated PTH increases calcium release by stimulating osteoclast activity. In addition, PTH stimulates and inhibits calcium and phosphate reabsorption in the kidney, respectively. These events ultimately can lead to hypercalcemia by more than 10.5 mg/dL, and hypophosphatemia less than 2.5 mg/dL [11]. In this regard, parathyroidectomy is well‐known as the gold standard and only curative treatment of GPA [12]. PHPT can manifest as osteoporosis, hypercalciuria, pancreatitis, kidney stones and nephrocalcinosis, gastrointestinal, cardiovascular symptoms, cognitive disorders, and vertebral fractures. Other clinical cases of PHPT, such as classic disease and normocalcaemic PHPT, are rare forms of PHPT. Early diagnosis is vital to prevent severe complications linked to hypercalcemia, with careful differentiation from parathyroid carcinoma being essential due to overlapping clinical features.

In this report, we present a case of a 37‐year‐old female patient with sporadic PHPT who presented to a pulmonologist for a check of cough and dyspnea and was diagnosed with GPA.

## 2 Case Presentation/Examination

**FIGURE 1:**
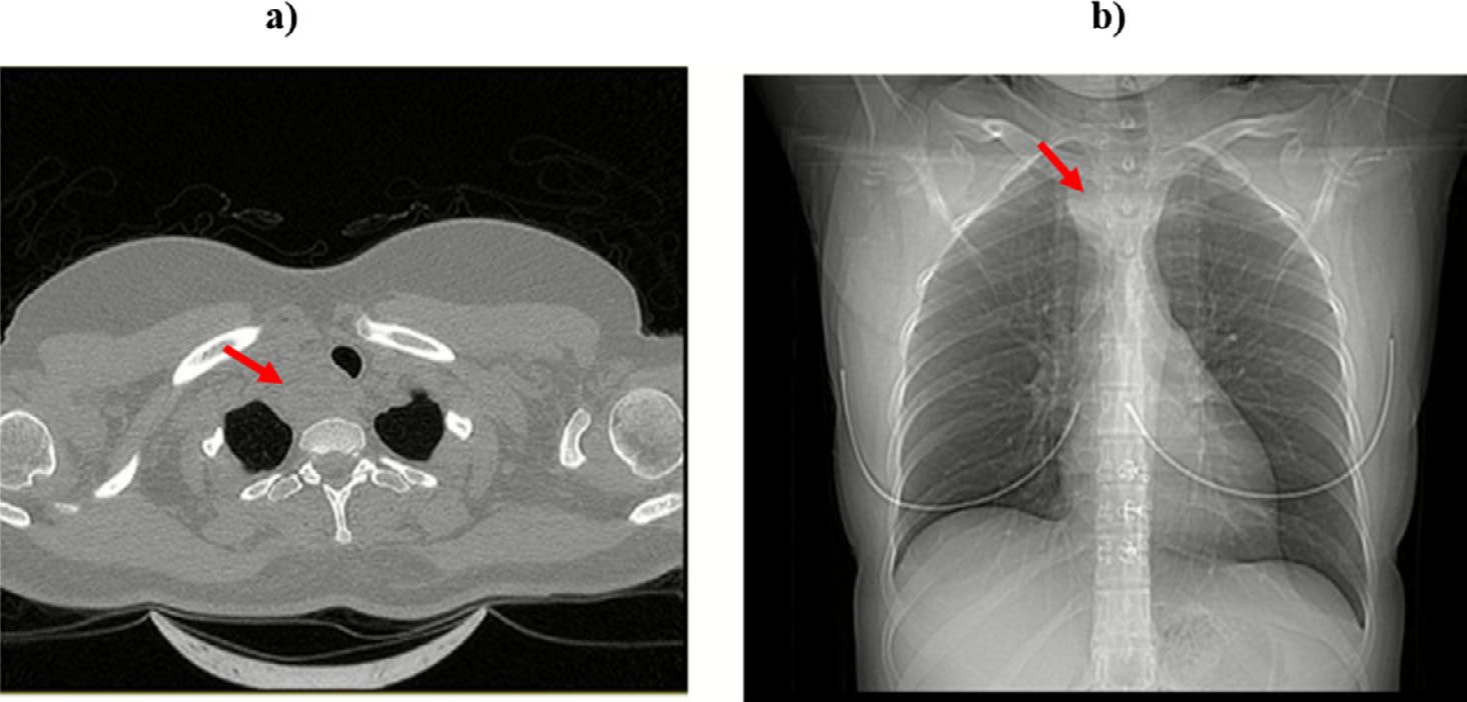
In the superior mediastinal cut, tracheal displacement is noted to the left anterior due to a right parathyroid adenoma. (a) Chest CT scan, and (b) scout view.

A 37‐year‐old woman presented to the pulmonology clinic with chronic cough and shortness of breath. The patient was evaluated by the pulmonologist and was found to have a nonproductive cough without fever, hemoptysis and weight loss. The patient experienced positional dyspnea, which typically took place in the supine position and was not connected to any activity. In addition, the dyspnea was not accompanied by pleuritic chest pain. In further investigations, it was found that she did not have fatigue, muscle weakness, bone pain, constipation, or polyuria. The patient's lungs were found clear to auscultation. The patient underwent a computed tomography (CT) and a hypodense mass was found in the superior mediastinum with compression of the trachea (Figure 1). Based on the results of CXR, mediastinal widening, and neck sonography, the patient was referred to our endocrine department. The patient did not experience exhaustion, muscle frailty, skeletal discomfort, irregular bowel movements, or excessive urination. During the endocrinologist's examination, it was revealed that the patient did not have concentration disorders and polydipsia, as well as a history of nephrolithiasis. On physical examination, it was found that she had an enlarged thyroid gland and a positive Pemberton's sign. The sonographic report showed a large solid‐cystic nodule measuring 27*30*70 mm and a volume of 27 cc, in the lower part of the RT lobe with extension to the retrosternal region and tracheal deviation, such that its solid component was isoechoic and hypovascular in color Doppler ultrasonography without any calcification. A fine‐needle aspirate (FNA) was done during ultrasound from the nodule, from which bloody fluid was flowing out during sampling. A cytological examination was performed on the specimen, where 20–40 follicular cells (suboptimal species) were observed on two slides, and the nodule was detected as a benign follicular nodule.

## 3 Methods: Differential Diagnosis, Investigations, and Treatment

**FIGURE 2:**
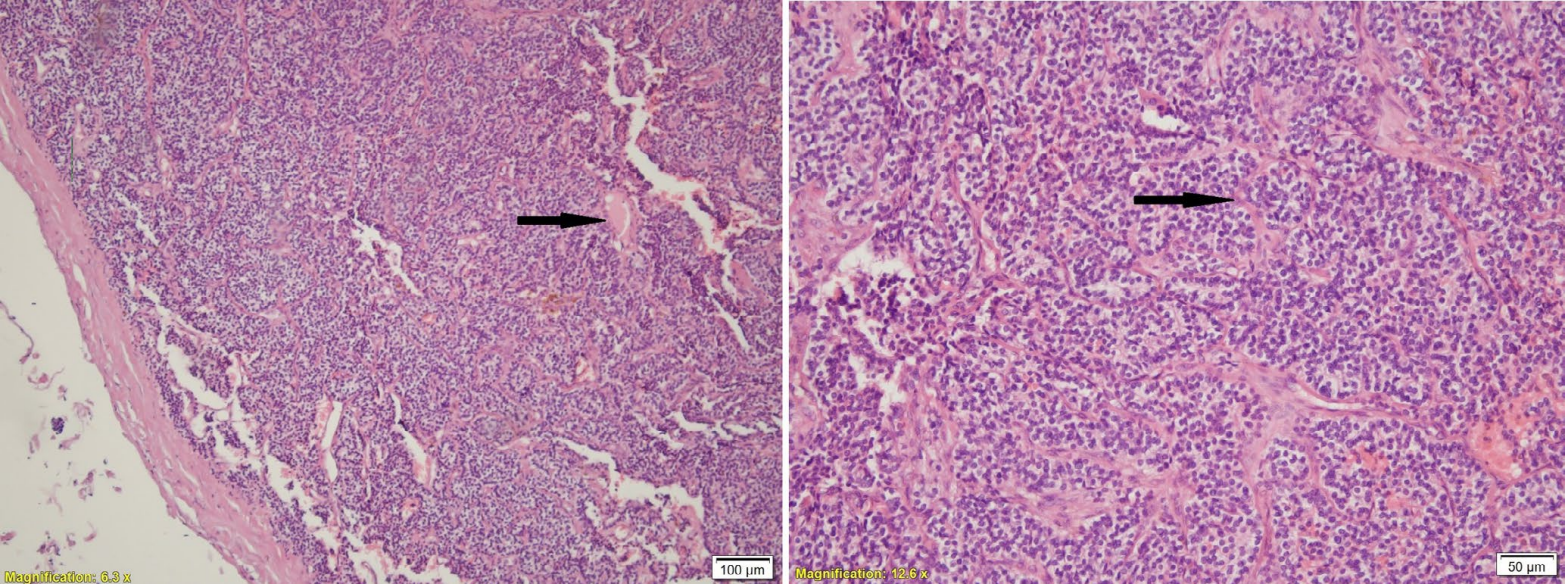
Histopathology confirmed the giant parathyroid adenoma (hematoxylin and eosin stain, 100X magnification). The lesion contains chief cells and water‐clear cells.

Finally, after extensive consultation with an endocrinologist and surgeon, the patient had the thyroid removed. Histopathological evaluation confirmed the presence of a parathyroid adenoma with cystic changes measuring 5 x 3 x 1 cm (weight 15 g) without atypia and vascular invasion (Figure 2). Two days after thyroidectomy, the patient returned to the hospital with symptoms of paresthesia around the mouth, fingers, and muscle cramps. Biochemical findings showed the serum levels of calcium and phosphorus were 8.5 mg/mL, and 2 mg/mL, respectively (Table 1). The patient was treated in the hospital and administered calcium gluconate and calcitriol.

**TABLE 1:** Results of biochemical tests of the patient.

Variable	Result	Units	Normal range
*Biochemical results*			
Calcium	8.5	mg/dl	8.6 to 10.3
Phosphorus (*p*)	2	mg/dl	2.8 to 4.5
Intact parathyroid hormone (iPTH)	13.5	pg/ml	10 to 55
Sodium (Na)	137	mg/dl	135 to 145
Potassium (K)	3.5	mg/dl	3.6 to 5.2
Magnesium (Mg)	1.4	mEq/L	1.3 to 2.1
Albumin	3.9	g/dl	3.4 to 5.4
Ca^+2^	1.04	mmol/L	1.12 to 0.32
*Blood gas results*			
pH	7.40	—	—
PCO_2_	38	mmHg	—
HCO_3_	22	mmol/L	—

## 4 Outcome and Follow‐Up

The calcium and iPTH levels normalized during the patient's follow‐up, and the patient's symptoms were resolved after treatment and recovered completely. The data associated with the levels of calcium before thyroidectomy is not shown.

## 5 Discussion

Parathyroid adenomas (PA) are a main cause of PHPT, and parathyroid carcinoma (PC) is the least common cause of PHP. In this regard, when adenomas exceed 2 cm in diameter, a precise distinction between GPA and parathyroid carcinoma should be performed using the depth/width ratio as a specific imaging parameter. A value of this image index below 1 indicates the presence of GPA in 94% of cases. In addition, serum PTH levels are associated with adenoma size (> 232 ng/L) [13]. According to the physiological function of the parathyroid gland, PTH may be the cause of elevated serum calcium, which increases bone resorption and decreases urinary calcium excretion [14]. In addition to hypercalcemia, GPA can cause anatomical symptoms including dyspnea and dysphagia. However, clinical reports indicate that hypercalcemia is one of the most common symptoms of parathyroid adenomas. PHPT caused by nongiant PA usually is accompanied by asymptomatic hypercalcemia and enhanced PTH, while according to studies, hypercalcemia and increased PTH are clearly detectable in GPA. These indicators are clearer and less symptomatic in the GPA. In 20% of adult patients, myalgia and fatigue, bone pain, arthralgia, constipation, kidney stones and psychiatric disorders are known as common symptoms. In addition to the biochemical tests, parathyroid pathology diagnosis, is performed through multiple methods such as radiologic imaging and biopsy.

This case of a 37‐year‐old female patient having cough and shortness of breath that were thought to be lung disease, while was diagnosed with a cervical neck mass that spread to the mediastinum and evidence of pressure on the trachea, and with the diagnosis of a thyroid nodule. Therefore, she was diagnosed as a surgical candidate due to the compressive symptoms observed. After the surgery, the patient had symptoms of hypocalcemia, including paresthesia around the mouth and carpopedal spasm. After further pathological investigations, it was found that this case had a giant parathyroid adenoma. Adenomas weighing more than 3.5 g are known as giant adenomas. Normally, with the increase in the size of the parathyroid adenoma, the amount of PTH also increases, and the patient becomes symptomatic. However, in some cases, due to bleeding and infarction inside the adenoma, some parts may be nonfunctional. In our case, an adenoma with a weight of 15 g was detected, and according to the acute onset of symptoms of cough and shortness of breath with no symptoms of hypercalcemia, these signs could be caused by the adenoma's size, along with the bleeding inside the adenoma and its compressive effect on the trachea. A limited number of studies in the past years have also reported cases similar to our patient with no symptoms and no hypercalcemia. For example, Haldar et al. (2014) reported a case of a giant parathyroid adenoma in a 61‐year‐old woman who used warfarin [15]. The authors excised it via an invasive transcervical method after radiological localization. The patient had recovered fully with biochemical resolution of hypercalcemia in six weeks [15]. In another report, Castro et al. (2017) described an asymptomatic case with a palpable nodule [16].

In addition, Al‐Hassan et al., in their literature review of GPA, 11 cases were reported with mediastinal and retrosternal masses, none of which described pressure symptoms [17]. In our case, 3 days after the surgery, the patient presented with paresthesia and carpopedal spasm symptoms. Despite the calcium level being in the minimum normal range, she was positive for Trousseau and Chvostek signs and had prolonged QTc: 410 msec, which can be caused by the downregulation of calcium receptors in secondary target organs. In addition, cytological examination indicated a follicular nodule with a small subset of cells demonstrating enlarged nuclei. A benign follicular nodule is a noncancerous lump in the thyroid gland. The risk of cancer in a benign follicular nodule is very low [18]. However, routine examinations are essential to observe any alterations. Should there be modifications in size or appearance, additional assessment may be required. In summary, the interesting findings in this patient were no high serum calcium level and other symptoms including fatigue, bone pain, constipation, muscle weakness, anorexia, nausea, and vomiting. Therefore, it is necessary to consider differential diagnosis for any neck mass, even if it is a sporadic tumor.

## 6 Conclusion

In conclusion, a giant parathyroid adenoma can present as a mediastinal or cervical mass with pressure symptoms, and hypercalcemia may not always occur in these patients. Therefore, nodules can be misdiagnosed as thyroid masses. The differential diagnosis of these cases must be done with precision and appropriate methods.

## Author Contributions

**Somayeh Chahkandi:** conceptualization, investigation, validation, writing – original draft, writing – review and editing. **Nasrin Amiri‐Dashatan:** data curation, methodology, writing – original draft, writing – review and editing. **Mehdi Koushki:** validation, writing – original draft, writing – review and editing. **Mojgan Sanjari:** conceptualization, investigation, supervision, validation.

## Funding

The authors have nothing to report.

## Ethics Statement

This case report did not require formal ethical approval, as it pertains to a single patient with anonymized data; written informed consent was acquired from the parents for publication, which includes the use of clinical images.

## Consent

Written informed consent was obtained from the patient for publication of this case report and any accompanying images.

## Conflicts of Interest

The authors declare no Conflicts of Interest.

## Data Availability

The data used to support the findings of this study are included within the article. Additional information can be requested by contacting the corresponding author.

## References

[1] T. Madkhali , A. Alhefdhi , H. Chen , and D. Elfenbein , “Primary Hyperparathyroidism,” Turkish Journal of Surgery 32, no. 1 (2016): 58–66, 10.5152/UCD.2015.3032. 26985167 PMC4771429

[2] S. T. Y. Htoo and N. E. Cusano , “Management of Primary Hyperparathyroidism: Historical and Contemporary Perspectives,” Endocrine Practice 31 (2025): 1488–1494, 10.1016/j.eprac.2025.07.009. 40683368

[3] M. W. Yeh , P. H. G. Ituarte , H. C. Zhou , et al., “Incidence and Prevalence of Primary Hyperparathyroidism in a Racially Mixed Population,” Journal of Clinical Endocrinology & Metabolism 98, no. 3 (2013): 1122–1129, 10.1210/jc.2012-4022. 23418315 PMC3590475

[4] M. Mizamtsidi , C. Nastos , G. Mastorakos , et al., “Diagnosis, Management, Histology and Genetics of Sporadic Primary Hyperparathyroidism: Old Knowledge With New Tricks,” Endocrine Connections 7, no. 2 (2018): R56–R68, 10.1530/EC-17-0283. 29330338 PMC5801557

[5] M. Beebeejaun , E. Chinnasamy , P. Wilson , A. Sharma , N. Beharry , and G. Bano , “Papillary Carcinoma of the Thyroid in Patients With Primary Hyperparathyroidism: Is There a Link?,” Medical Hypotheses 103 (2017): 100–104, 10.1016/j.mehy.2017.04.016. 28571792

[6] N. G. Mokrysheva , A. K. Eremkina , S. S. Mirnaya , et al., “The Clinical Practice Guidelines for Primary Hyperparathyroidism, Short Version,” Problems of Endocrinology 67, no. 4 (2021): 94–124, 10.14341/probl12801. 34533017 PMC9753843

[7] F. Marini , L. Cianferotti , F. Giusti , et al., “Molecular Genetics in Primary Hyperparathyroidism: The Role of Genetic Tests in Differential Diagnosis, Disease Prevention Strategy, and Therapeutic Planning. A 2017 Update,” Clinical Cases in Mineral and Bone Metabolism 14, no. 1 (2017): 60–70, 10.11138/ccmbm/2017.14.1.060. 28740527 PMC5505716

[8] D. M. Press , A. E. Siperstein , E. Berber , et al., “The Prevalence of Undiagnosed and Unrecognized Primary Hyperparathyroidism: A Population‐Based Analysis From the Electronic Medical Record,” Surgery 154, no. 6 (2013): 1232–1238, 10.1016/j.surg.2013.06.051. 24383100

[9] S. Shah , P. Fujikawa , K. Brand , V. Munshi , and K. Patel , “Giant Parathyroid Adenoma: A Case Report,” Cureus 15, no. 1 (2023): e34140, 10.7759/cureus.34140. 36843787 PMC9948681

[10] S. Rutledge , M. Harrison , M. O'Connell , et al., “Acute Presentation of a Giant Intrathyroidal Parathyroid Adenoma: A Case Report,” Journal of Medical Case Reports 10, no. 1 (2016): 1–6, 10.1186/s13256-016-1078-1. 27756436 PMC5070099

[11] P. M. Spanheimer , A. J. Stoltze , J. R. Howe , S. L. Sugg , G. Lal , and R. J. Weigel , “Do Giant Parathyroid Adenomas Represent a Distinct Clinical Entity?,” Surgery 154, no. 4 (2013): 714–719, 10.1016/j.surg.2013.05.013. 23978594 PMC3787983

[12] G. Ghilardi and L. De. Pasquale , “Hungry Bone Syndrome After Parathyroidectomy for Primary Hyperthyroidism,” Surgery Current Research 4, no. 2 (2014): 1–5, 10.4172/2161-1076.1000168.

[13] D. Calva‐Cerqueira , B. J. Smith , M. L. Hostetler , et al., “Minimally Invasive Parathyroidectomy and Preoperative MIBI Scans: Correlation of Gland Weight and Preoperative PTH,” Journal of the American College of Surgeons 205, no. 4 (2007): S38–S44, 10.1016/j.jamcollsurg.2007.06.322. 17916517

[14] D. Goltzman , “Physiology of Parathyroid Hormone,” Endocrinology and Metabolism Clinics 47, no. 4 (2018): 743–758, 10.1016/j.ecl.2018.07.003. 30390810

[15] A. Haldar , A. Thapar , S. Khan , and S. Jenkins , “Day‐Case Minimally Invasive Excision of a Giant Mediastinal Parathyroid Adenoma,” Annals of the Royal College of Surgeons of England 96, no. 5 (2014): e21–e23, 10.1308/003588414X13946184900480. PMC4473961 24992407

[16] M. A. Castro , A. A. López , L. M. Fragueiro , M. Araujo Castro , and N. P. García , “Giant Parathyroid Adenoma: Differential Aspec.Ts Compared to Parathyroid Carcinoma,” Endocrinology, Diabetes & Metabolism Case Reports 2017 (2017): 17–0041, 10.1530/EDM-17-0041. PMC5420060 28491324

[17] M. S. Al‐Hassan , M. Mekhaimar , W. El Ansari , et al., “Giant Parathyroid Adenoma: A Case Report and Review of the Literature,” Journal of Medical Case Reports 13, no. 332 (2019): 1–9, 10.1186/s13256-019-2257-7. 31722742 PMC6854700

[18] G. Grani , M. Sponziello , S. Filetti , and C. Durante , “Thyroid Nodules: Diagnosis and Management,” Nature Reviews Endocrinology 20, no. 12 (2024): 715–728, 10.1038/s41574-024-01025-4. 39152228

